# Checking Contact Tracing App Implementations with Bespoke Static Analysis

**DOI:** 10.1007/s42979-022-01357-w

**Published:** 2022-09-28

**Authors:** Robert Flood, Sheung Chi Chan, Wei Chen, David Aspinall

**Affiliations:** 1 LFCS, University of Edinburgh, Edinburgh, Scotland, UK; 2 MACS, Heriot-Watt University, Edinburgh, Scotland, UK; 3 Alan Turing Institute, London, England, UK

**Keywords:** Static analysis, COVID-19, Contact tracing, Android, MonSTER

## Abstract

In the wake of the COVID-19 pandemic, contact tracing apps have been developed based on digital contact tracing frameworks. These allow developers to build privacy-conscious apps that detect whether an infected individual is in close proximity with others. Given the urgency of the problem, these apps have been developed at an accelerated rate with a brief testing period. Such quick development may have led to mistakes in the apps’ implementations, resulting in problems with their functionality, privacy and security. To mitigate these concerns, we develop and apply a methodology for evaluating the functionality, privacy and security of Android apps using the Google/Apple Exposure Notification API. This is a three-pronged approach consisting of a manual analysis, general static analysis and a bespoke static analysis, using a tool we have developed, dubbed MonSTER. As a result, we have found that, although most apps met the basic standards outlined by Google/Apple, there are issues with the functionality of some of these apps that could impact user safety.

## Introduction

As governments around the world attempt to contain the spread of the COVID-19 virus, the research area of digital contact tracing has grown rapidly with several methods being proposed to aid this cause. Digital contact tracing refers to the tracking of individuals to determine potential exposure between an infected patient and a user, using mobile technologies such as QR codes, Bluetooth and GPS. Currently, the most effective strategy to control the outbreak is widespread social-distancing and isolation of even healthy individuals. This has significantly impacted almost every aspect of daily life, with profound economic and social drawbacks. Health authorities hope digital contact tracing will allow for social-distancing measures to be eased by automating the time-consuming process of manual contact tracing, allowing more individuals to discover whether they are infected.

Such efforts may leave users vulnerable to security and privacy flaws. Due to the urgency in developing contact tracing apps, many have been built at an accelerated rate. It is unclear if measures were undertaken to minimise the risk of security vulnerabilities. As these apps need to be used by a large segment of a country’s population to be effective, the integrity of many people’s data and digital assets may be at risk.

So far, ensuring tracing frameworks maintain strong privacy guarantees has been the focus of research, with new privacy-preserving frameworks being designed by several parties [1–3]. However, little research has investigated implementations of these frameworks; the apps using these frameworks can violate these privacy guarantees by sharing additional information. As digital contact tracing techniques involve the collection of sensitive medical and location data to function, there are severe privacy implications if this information is improperly handled. For instance, the Bahraini *BeAware* was linked to a televised game-show ‘Are You At Home?’, where users of the app were called and offered prizes if they were at home and shamed otherwise. This shows leveraged data collected from the app such as the contestant’s name and phone number [4]. Such misuse could have been prevented using a privacy-preserving framework, provided that the app did not retrieve further information. Thus, it is important that a tracing app uses a privacy-preserving framework, but also it does not share data beyond what the framework allows. As government bodies urge people to use tracing apps, they are an enticing target for malicious actors. Malware claiming to be contact tracing software or malicious, repackaged versions of contact tracing apps exist [5, 6] and more are likely to be discovered. Developers may also introduce security problems via misuse of these frameworks. Therefore, tracing frameworks have considered potential security issues in their design. For instance, the Google/Apple Exposure Notifications (GAEN) API acts as a security boundary, allowing developers to access its functionality whilst shielding them from its internal operation. However, previous similar secure APIs have had faulty implementations that led to fundamental issues, such as the PCKS 11 API of hardware security modules leaking their private keys [7]. This paper introduces a methodology for analysing the functionality, privacy and security of COVID-19 contact tracing apps. We employ manual and static analysis alongside a bespoke, customisable static analysis tool. This tool, developed by us and dubbed MonSTER, ensures apps adhere to the requirements of a given framework, something impossible with an out-of-the-box tool, and can provide repeatable, lightweight checks throughout an app’s development and updates. We target apps using the GAEN API. Our approach and analysis focus solely on the implementation of the apps themselves and assume the adopted contact tracing framework is well defined and problem-free. This helps protect against mistakes by app developers, as well as attacks and infiltration by adversaries by discovering potential security vulnerabilities. Ultimately, work like this may help assuage public concerns of using these apps and increase uptake, helping to better contain the spread of COVID-19. The contributions of this paper are as follows:

- We design a methodology with three stages, manual, off-the-shelf static, and bespoke static analysis, to evaluate the functionality, privacy and security of contact tracing apps.
- We develop MonSTER, a configurable, lightweight static analysis tool to verify an app’s adherence to API usage requirements. We have made this tool publicly available.^1^
- We collect a set of 12 contact tracing apps using the GAEN API and, where necessary, rebuild the apps from their source code to obtain a non-obfuscated APK. We collect two versions of each app, from 2020 and 2021 to chart their development over time. We have released these apps as a public dataset.^2^
- We obtain results demonstrating that, although the majority of apps tested functioned correctly, there were implementation problems in some apps that impacted their functionality. Namely, we found that apps may incorrectly inform users contact tracing is enabled when it is in fact disabled during reasonable usage.

The structure of the rest of this paper is as follows. The section “Background” provides background information, including security and privacy concerns, and an overview of the GAEN API. The section “Methodology” describes our methodology, including our lightweight static analysis tool *MonSTER* (*Mon*oid-based *St*atic Analys*er*). The section “MonSTER Checks” describes the process we undertake for our manual, static, and MonSTER analysis in more detail. The section “Results” shows the results of this analysis. The section “Limitations” discusses the limitations of our approach. The section “Related Work” summarises the related work. The section “Retrospective and Conclusion” concludes with a retrospective view of the contact tracing app landscape and discusses ongoing and future steps.

## Background

Due to privacy concerns surrounding the collection of user location data by governments, decentralised approaches to digital contact tracing are promoted. Decentralised approaches trace contacts with minimal interaction with a central database; several have been proposed, including *TCN* [8], *DP3T* [2] and the *Google/Apple Exposure Notification* protocol or GAEN for short [1]. They are similar in design, using Bluetooth Low Energy (BLE) to measure distance between users. The effectiveness of BLE tracking has been criticised [9], but tracking can be augmented with the use of QR-code registrations, and even a partially successful approach may help. In this section, we introduce several existing contact tracing methodologies and discuss details of the GAEN Framework and potential security/privacy problems.

### Contact Tracing Methodologies

Recently, increasing numbers of researchers and industry leaders have started to raise awareness of the security and privacy concerns of contact tracing apps. To balance the effectiveness of contact tracing methodologies with the security, privacy and anonymity of app users, they began to design both open-source and proprietary frameworks to handle the sensitive aspects of digital contact tracing. These frameworks take responsibility in handling the sensitive information and only provide developers with access to the necessary functions to implement apps. These abstractions of these sensitive features allow the app developers to focus solely on their development and not on the underlying contact tracing methodology; they just need to follow the security guidelines and implement the function/method calls accordingly without the need to consider serious security problems. In this paper, we are not considering the security features of those underlying contact tracing framework developed by those researchers and industry leaders. We are instead considering if the app developers implement and use the data and method calls of the contact tracing framework securely and follow the security and functionality requirements of these open-sourced frameworks.

There are currently several dozen contact tracing apps adopting and employing a wide range of contact tracing frameworks and methodologies. As more users and developers are concerned about the security and privacy issues, many of these apps choose to adopt one of the many recommended privacy-preserving protocols designed by researchers and industry leaders. Example includes the Decentralised Privacy-Preserving Proximity Tracing (DP3T) [2], Google/Apple Exposure Notification [1] or the Pan-European Privacy-Preserving Proximity Tracing [10] (PEPP-PT) specification. On the other hand, some apps still consider using their own proprietary methodologies which are not the concern of this paper.

The major features of these contact tracing methodologies and frameworks differ on how to determine proximity between users and how to store and identify whether a user is in close proximity with an individual who is diagnosed with the virus. All of these frameworks implement a different combinations of these features. For proximity determination, some of them make use of Bluetooth Low Energy (BLE), GPS location service or active QR-code scanning. On the other hand, the storage of information for contact tracing is generally divided into two approaches: one uses a centralised server to store contact and infection information, whilst the other uses a decentralised approach where most of the information is stored in the user’s device. These different storage approaches directly affect how, where and what data should be protected during the whole contact tracing period.

**Fig. 1:**
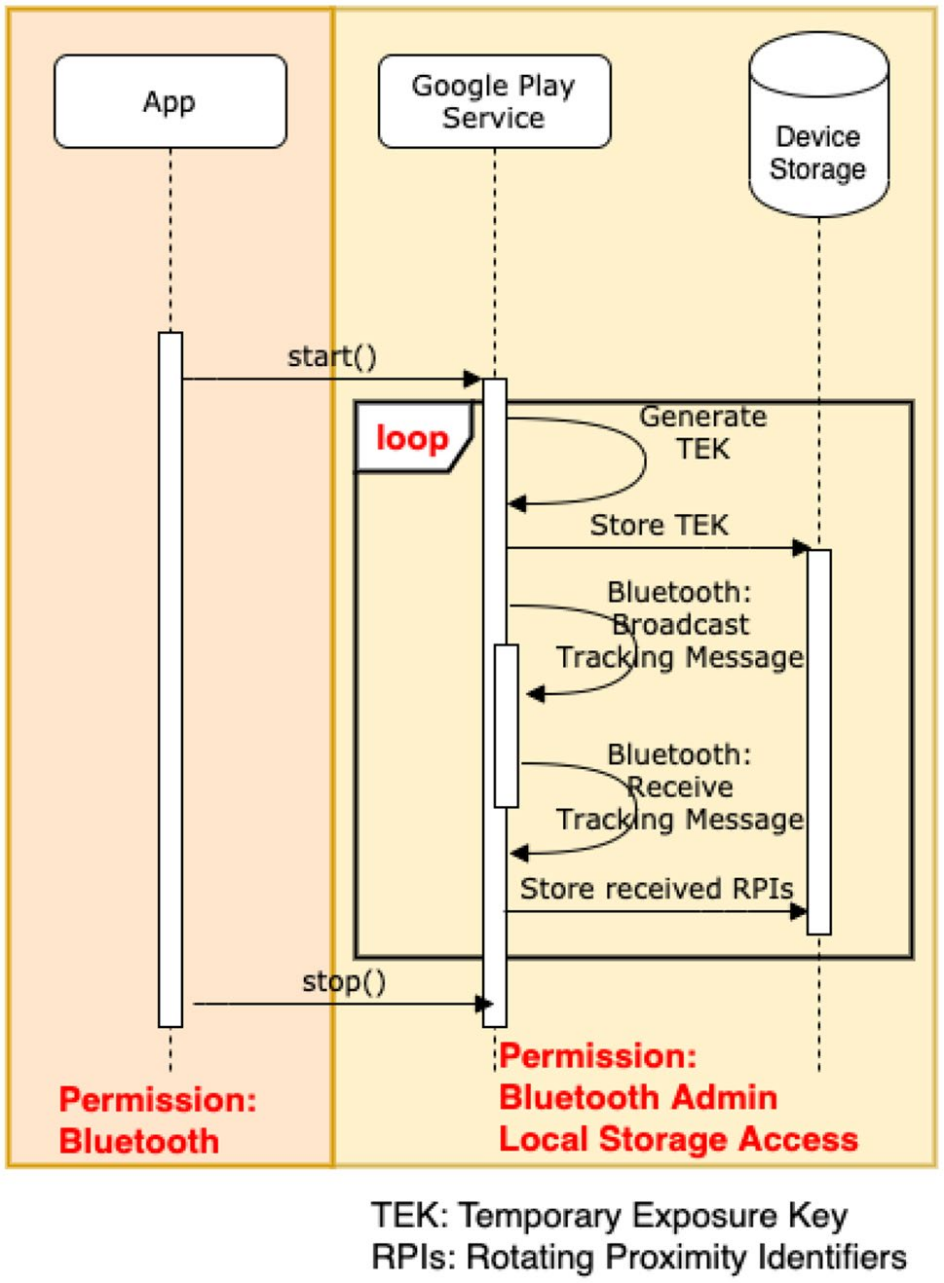
Contact exchange

**Fig. 2:**
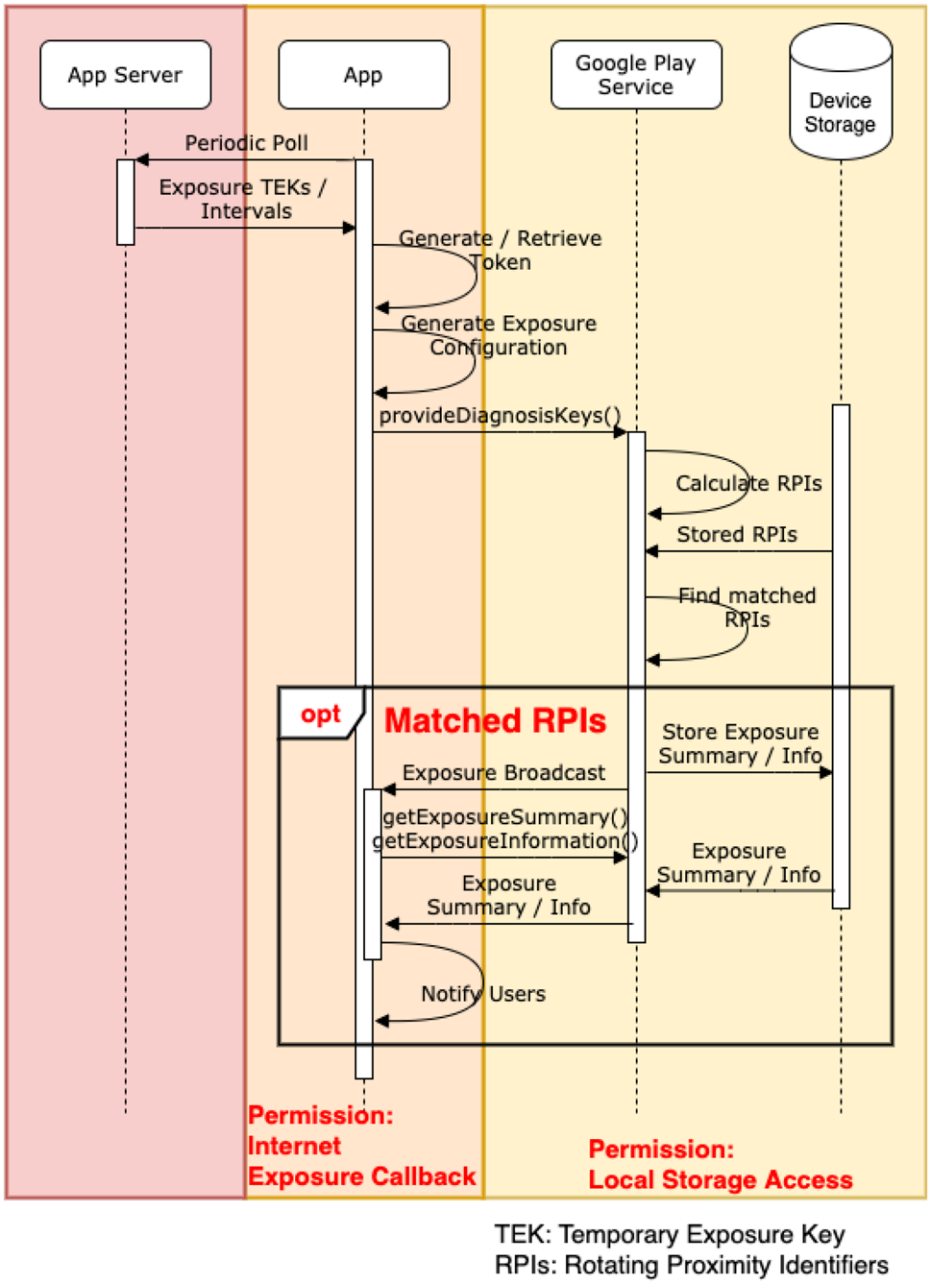
Exposure update

### Google/Apple Exposure Notification

This framework is developed by Apple and Google for their mobile operating systems, each providing the same set of API calls. The major features of the GAEN Framework are divided into three stages. For each stage, we summarise the responsibilities of the app developers, the communication of the servers and the underlying APIs. We focus our research on version 1.3 of the framework.

*Contact Exchange* This feature makes use of BLE with keys and identifiers—unique, random numbers derived from the keys—that are modified frequently. These keys and identifiers serve as a record of all people that a user has been in close proximity with over the last 14 days. The framework handles rotating, generating and exchanging all keys and identifiers. The app’s responsibility is limited to providing the ability to start/stop the service. All keys and identifiers exchanged will be stored locally and the app should not have access to them. When first setting up the app, the device generates a *Temporary Exposure Key* (TEK) using a cryptographically secure random number generator. Every 24 h, a new TEK is generated. Two further keys are generated using this key via HKDF, a *Rolling Proximity Identifier Key* and an *Associated Encrypted Metadata Key*. These keys are in turn used to generate two BLE payloads: the *Rolling Proximity Identifier* (RPI) and the *Associated Encrypted Metadata* (AEM). These consist of a byte string acting as an identifier and a payload that can be later decrypted to reveal the user’s TEK, both encrypted using AES-128. The phone alternates between the Bluetooth client and host modes, continuously broadcasting the RPI and AEM when in client mode and seeking such broadcasts from nearby phones when in host mode, storing any received payloads. Figure 1 shows a sequence diagram for this stage.

*Exposure Update* All identifiers are all anonymous and should not be linked with a real identity. It is the responsibility of the server to only store this information without recording which device uploaded them. Also, as mentioned above, these identifiers received from other devices through Bluetooth are handled by the underlying framework and the apps cannot access them as they are stored in protected storage locations. The apps are responsible to poll the central server periodically to retrieve the new list of infected identifiers and pass it to the framework. The framework will match the stored identifiers with the infected list and determine if the user has been in contact with an infected person. If the result is positive, the framework will send a broadcast message of the infection. The app should implement a broadcast receiver to receive this information and use the API call to retrieve further exposure summary information, notifying the users of this fact. None of these steps should reveal the identity of the infected person nor the notified users. To determine if a user was exposed to an infected person, users routinely download the list of newly added Diagnosis Keys from the Diagnosis Server. As RPIs are derived from TEKs, each client can then derive a series of RPIs from these Diagnosis Keys. These derived identifiers can then be matched with the list of stored identifiers discovered over BLE scanning. If any of the derived identifiers match a stored identifier, then the user has come into contact with someone infected with COVID-19 and the app should notify them of this. The app should implement a broadcast receiver to receive this information and use an API call to retrieve further exposure summary information, notifying the users of this fact. None of these steps should reveal the identity of the infected person nor the notified users. Figure 2 shows a sequence diagram for this stage.

**Fig. 3:**
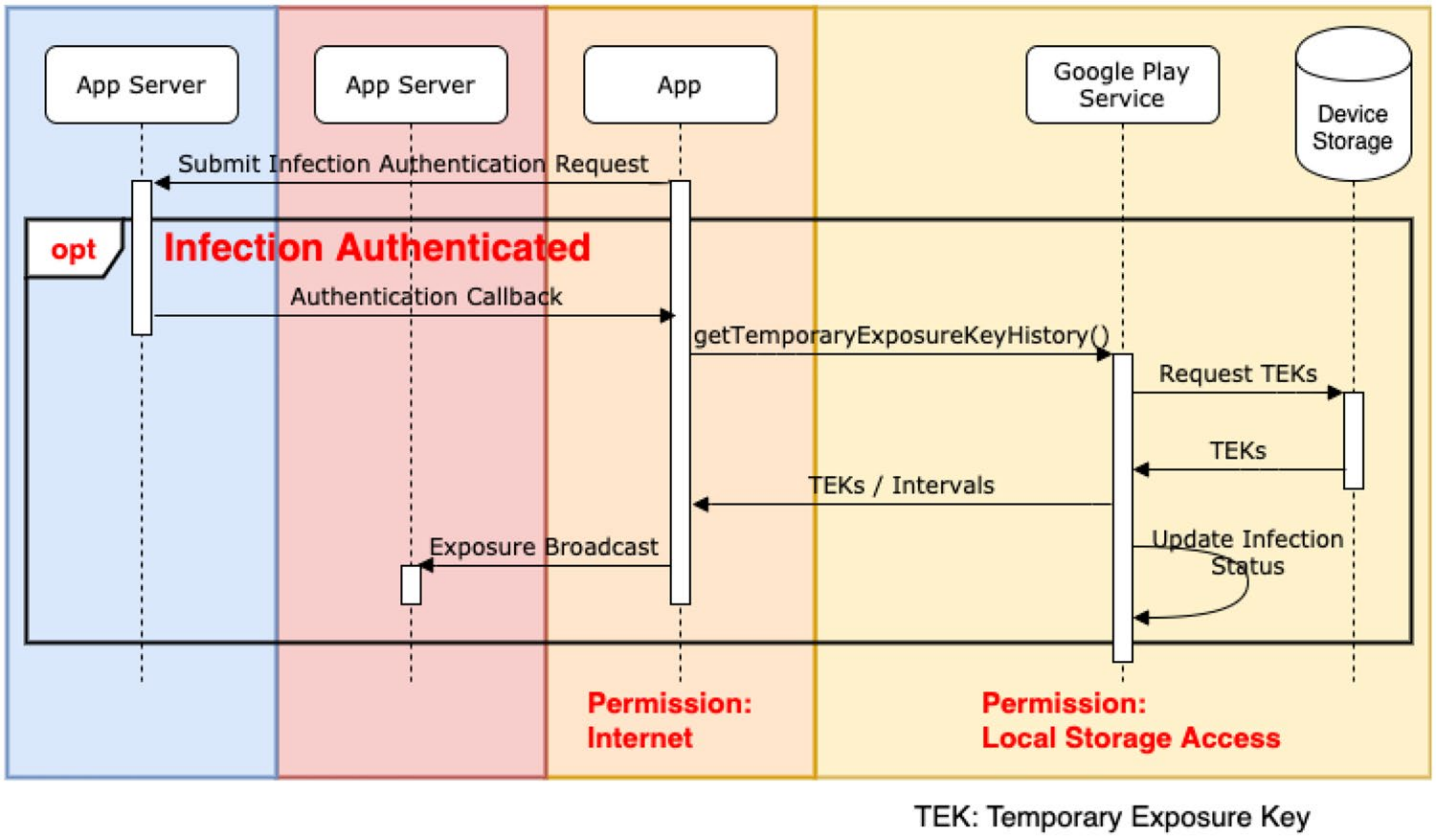
Infection report sequence diagram

*Infection Report* If a user tests positive with COVID-19, they can choose to upload their TEK history, extending back 14 days, to a *Diagnosis Server*, alongside a timestamp to describe when their validity started. These are referred to as *Diagnosis Keys* and only leave the device if the user tests positive. The contact tracing app is responsible for verifying a diagnosis report from an authorised medical provider. The apps are responsible for protecting this information during the uploading process. Identifiers over 14 days old are considered no longer useful as infected user would have likely recovered in that time. Thus, identifiers are deleted from both the device and central server 14 days after being first created. Figure 3 shows a sequence diagram for this stage.

#### App Responsibilities

To aid the development of these apps, Google and Apple provided the GAEN API and documentation outlining its usage. They describe the functionality the API provides and the functionality app developers must provide. The API handles the complex aspects of the protocol including the cryptographic systems, the broadcasting and collection of BLE data and the calculation of an ‘exposure risk level’. The documentation states the app must:

1. Allow users to start and stop contact tracing.
2. Register a Broadcast Receiver to receive the ACTION_EXPOSURE_STATE_UPDATED intent
3. Poll the Diagnosis server to obtain keys.
4. Download Diagnosis Keys and provide them to the API.
5. Upload TEKs after a positive test and the user has provided permission.
6. Notify the user with medical information when they have been exposed to an infected user.

These responsibilities are part of the API lifecycle outlined in the section “Google/Apple Exposure Notification”. To fulfill these, the GAEN Framework provides developers with an API via the ExposureNotificationClient class. In this paper, we focus on the API methods start, stop, isEnabled, getTemporaryExposureKeyHistory (which retrieves the past 2 weeks of TEKs) and provideDiagnosisKeys (which submits downloaded keys to the API).

### Functionality Concerns

In developing our methodology for analysing the security, privacy and functionality of contact tracing apps, we consider two attacker models.

For the first, we assume the developers attempted to securely and faithfully adopt the GAEN framework but failed, reducing user privacy or providing exploit vectors. Although the GAEN API has been designed with privacy and security in mind, apps using this API must adhere to strict requirements to maintain these properties. For instance, developers accidentally misusing the GAEN API may build an app that retrieves and uploads the users’ TEK history overly frequently. This results in the online database resembling a centralised protocol, such as BlueTrace, damaging user privacy. These functionality failings may lead to numerous security and privacy violations: exposure of a user’s identity; their address; their infection status; the identities of their contacts; their location and the loss of security of their device, which may lead to other potential data exposures. Even if the GAEN API is correctly utilised, the app may contain vulnerabilities impacting user security, such as misconfigured webviews or exported components.

For the second, we consider the case where the app is malware purporting to perform contact tracing, perhaps as a repackaged version of a legitimate app. Such apps have already been discovered, including backdoors [5] and ransomware [6]. In this situation, the fact that the malware claims to be a contact tracing app is incidental to its true behaviour. Nevertheless, any systematic review of the security of contact tracing apps needs to consider this possibility.

There is much work considering the security and privacy properties of the GAEN framework, discussed in the section “Related Work”. Unlike this work, we treat the GAEN framework itself as a blackbox and assume it is functional and effective. This is reflected in the above attack models as we focus primarily on whether these apps have successfully adhered to the GAEN’s requirements. Without this assurance, the end-user has little guarantee that they are benefiting from the claimed protection of the GAEN framework, regardless of its effectiveness.

We discuss how these models inform our methodology for analysing contact tracing apps in the section “Methodology”.

### Static Analysis Tools

There are many static analysis tools for Android software. However, these tools know little about the context in which they are applied. Many tools used for Android code analysis are primarily Java analysis tools, such as Error-Prone [11] and FindBugs [12], and are unaware of any potential problems specific to the Android platform or any particular details of the app. Many security vulnerabilities are the result of faulty implementation logic which cannot be divorced from the app’s utility. As such, there are a wide range of bugs that general static analysis tools are incapable of finding.

Contact tracing apps are one domain where general static analysis is lacking and implementation bugs may arise [13]. Prior to March 2020, there were no tracing apps developers could base their apps on, and disparate teams are developing apps with little guidance. The challenges faced by tracing apps are unique, and it is unlikely general static analysis solutions could detect functionality issues or missing features, such as notifying the user when an exposure occurs. It would be extremely beneficial to have a static analysis tool that ensures an app is adhering to necessary standards during its development and release.

## Methodology

In this section, we discuss our methodology, together with the static analysis tool *MonSTER* (*Mon*oid-based *St*atic Analys*er*) developed to identify problematic patterns in Android apps which may pose security, privacy or functionality concerns. In this paper, we use this methodology to verify the apps studied in the section “Collection of Apps” adhere to the basic requirements needed to function as contact tracing tools, based on the responsibilities discussed in the section “App Responsibilities”.

Although we apply this three-pronged methodology, consisting of manual, general static and bespoke static analysis, to contact tracing apps, we stress this methodology is highly customisable and can be applied to many domains. The manual analysis acts as a research stage, providing us with an understanding of the inner workings of a set of related apps. During the general static analysis stage, we screen the apps for common vulnerabilities that could occur in any domain. We also ensure that the app is not malware. We then apply the knowledge gained during our manual analysis to our bespoke analysis stage, allowing us to search for design vulnerabilities that are unique to the domain in a repeatable, automated manner.

### Collection of Apps

We chose a set of Android apps using the GAEN framework with open-source code.^3^ Table 1 shows a list of the apps, their country of origin or developer, the analysed versions, the primary language used and the size of the code. We consider two different versions of each app. The first of these were downloaded on the 28th July 2020, except for *Stop-COVID-19* and *NHS Test & Trace*, which were downloaded on the 12th August 2020, and *Protect Scotland*, which was downloaded on the 10th September 2020. Only *SwissCovid* was developed using the DP3T protocol: this is extremely similar to the GAEN framework and uses the GAEN API as part of its design. We had to build some apps from source and disable ProGuard to generate unobfuscated APK files. This is the first ‘round’ of our analysis. We then repeat this process for the versions of the apps available on 25th August 2021 to evaluate their development over time, which is the second ‘round’ of our analysis. CovidShield and Covid Safe Paths were not updated during this period of time.

**Table 1:** Contact tracing apps

App name	Origin	Versions	Versions	Language	Code size	Code size
		(2020)	(2021)		(2020)	(2021)
ApturiCovid	Latvia	1.0.47	1.0.52	Kotlin	313 KB	360 KB
Corona-Warn-App	Germany	1.0	2.7.1	Kotlin	650 KB	4.7 MB
Covid Safe Paths	MIT	None	None	TypeScript	2.4 MB	2.4 MB
CovidShield	Shopify	None	None	TypeScript	790 KB	790 KB
Covid Tracker App	Ireland	1.0.4	1.0.7	TypeScript	430 KB	565 KB
Immuni	Italy	1.0.3	2.5.3	Kotlin	850 KB	935 KB
NHS Test & Trace	UK	3.0	4.10	Kotlin	570 KB	2 MB
Protect Scotland	Scotland	1.0.0.30	1.2.3	TypeScript	Unknown	425 KB
ProtegoSafe	Poland	1.0	4.12	Kotlin	500 KB	640 KB
Stop-Covid-19	Croatia	1.0	2.20	Java	230 KB	235 KB
Stopp Corona	Austria	1.2.0	2.1.4	Kotlin	860 KB	1.2 MB
SwissCovid (DP3T)	Swiss	1.0.4	2.0.1	Java/Kotlin	520 KB	680 KB

### Manual Analysis

Having collected a series of apps, we began a manual analysis process. First, we ran the apps and systematically iterated over all possible functionality, with the exception of the later stages of the key submission process. Following this, we began a code review, plotting out the general structure of each app and noting how they interacted with the GAEN API. We achieved this by finding where the app calls the various GAEN API methods and following their respective call flows through the application. Finally, we reviewed any publicly released documentation.

### General Static Analysis

We evaluated several Android static analysis tools as options for conducting off-the-shelf static analysis as might be used by a professional security analyst or penetration tester. Many tools are available freely and as commercial products; our point was to select something typical which demonstrates the capability of general static analysis tools in the context of our methodology, rather than find some “ultimate” best possible tool. We looked on GitHub and considered popularity as measured by GitHub stars. The most popular tools were MobSF [14] and QARK [15]. Ultimately, we chose MobSF, since QARK flagged many trivial issues.

MobSF flags many generic Android security problems, such as certificate issues, hard-coded API keys and blacklisted malicious domains. It provides a useful condensed overview of the app including measurements such as the permissions used, the included native code libraries and the number of components—including exported components which extend an app’s attack surface. Finally, MobSF summarises the overall code quality with an app security score, ranging from 0 to 100. We ran MobSF on all of our apps; the results are summarised in the section “General Static Analysis”.

### Bespoke Static Analysis: MonSTER

**Fig. 4:**
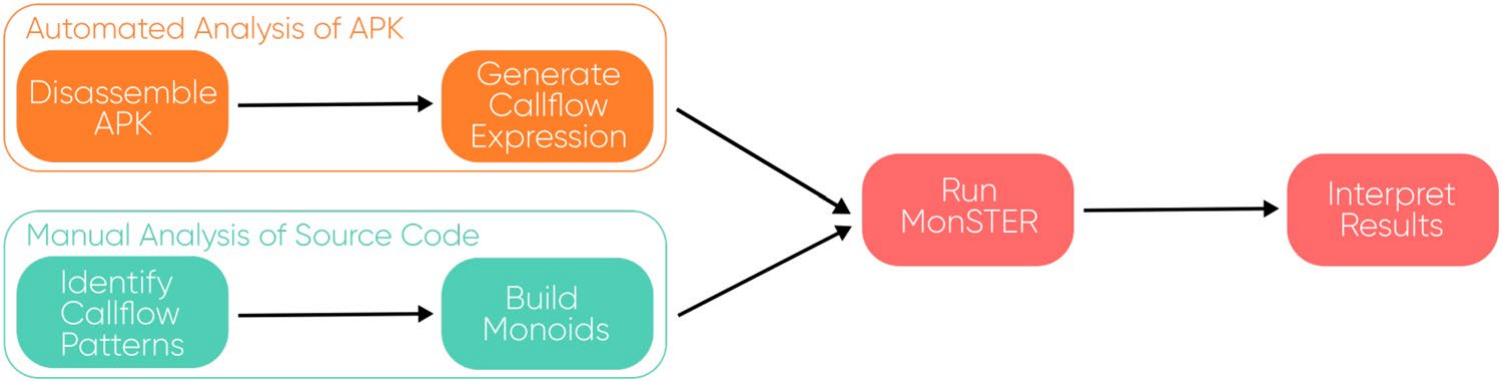
MonSTER workflow

MonSTER is a static analysis tool written in Haskell and Python, using Androguard [16]. It can be configured to detect patterns of method calls in Android apps. These patterns are customisable to ensure certain liveness and safety properties are present in an app. It is intended to function as a tool to aid the testing of apps during and after development, using bespoke patterns to detect desirable or undesirable properties (Fig. 4).

MonSTER uses a control-flow abstraction. A program is a collection of recursive procedures $f = e_f$ where *f* is a procedure identifier and $e_f$ is an expression in the grammar:1Here, *a* is an atomic procedure, and ;  and ? are sequential composition and non-deterministic branching.

MonSTER converts method calls from Dalvik bytecode in an APK into this expression language. Branch points are abstracted by considering exit points of basic blocks as potential branches. Methods whose body can be ignored—such as API calls or those we aren’t interested in—are treated as atomic methods and the rest are identifiers with definitions. This gives a set of expressions defining the overall program’s execution, called the *call flow expression form* of the APK. Methods from this expression form are lifted into a monoid, picking out ones of interest. 
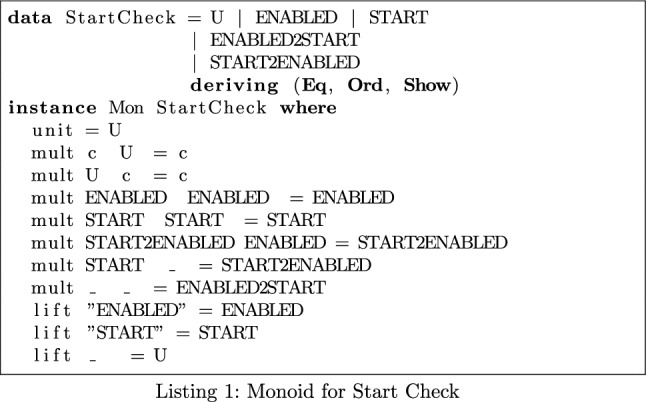


Consider the first GAEN check as an illustration. For many of the analysed apps, before starting the GAEN client, there is a check to see it is already running. If it is, the function exits gracefully. The GAEN documentation is unclear as to what happens when the API is started if already running and whether this causes unexpected behaviour, such as the “resetting” of the protocol, inhibiting its effectiveness. Therefore, we treat the already-running check as good practice and will use MonSTER to verify that it takes place.

We wish to verify that a call to isEnabled() is followed by a call to start(). In Listing 1, the monoid’s operator mult models method sequencing. To keep the monoid’s policy clear and succinct, we use *keywords* to stand for groups of methods—for instance, every method considered to be a network sink is modelled by **Sink**. In this case, we replace all appearances of the isEnabled() and start() methods with the keywords **Enabled** and **START**. When parsing the call flow expression, these keywords are embedded into the monoid using the lift operator shown in Listing 1. Methods that are not of interest are lifted to the monoid’s identity.

By creating such policies as monoids, we can define desirable or undesirable patterns customised to an app or app type. This is particularly relevant in the case of Contact Tracing Apps which rely on a common API. The expected behaviour of the GAEN can be encoded in this policy format, allowing MonSTER to perform checks specific to the Contact Tracing Domain. MonSTER is unique in this regard as such specificity is impossible with off-the-shelf, general static analysis software as they are geared towards detecting vulnerabilities irrespective of the target app’s functionality.

Once translated into the monoid, MonSTER generates a system of equations using the program’s call flow expression, consisting of a type expression for each procedure. MonSTER solves this system of equations by calculating its least fixed point. We expect to see the element **ENABLED2START** appear in the output of MonSTER only in cases where this pattern occurs.

MonSTER is kept simple by design: it focuses on an app’s call flow and ignores its data flow. However, crucially, it can capture call flows for continually executing code in its model of mutually recursive Büchi automata. Therefore, we can model the Android activity lifecycle, including implicit invocations (e.g., onCreate() followed by onStart()) as well as cycles (onStop() followed by onResume() then onStart() again). Liveness and safety properties can be ensured regardless of how the app is used, which is useful for checking longer term API call sequences such as used in GAEN. As the section “Introduction” mentioned, secure APIs can be vulnerable to API fuzzing attacks: a string of API calls in a certain order leads to a security issue. Such vulnerabilities, once discovered, could be captured as custom rules in MonSTER.

## MonSTER Checks

In this section, we outline the call flow patterns we aim to discover, alongside the example in the section “Bespoke Static Analysis: MonSTER”. These patterns are intended to act as sanity checks, allowing a developer to verify an app meets the API requirements and is functional. Although we apply these checks to contact tracing apps, the methodology is highly customisable and similar checks can be performed on a variety of apps. We base these checks around the necessary operations discussed in the section “App Responsibilities”. To ensure accuracy, we also test a modified version of the Google reference app^4^ designed to fail all of the checks.

### Registration of Broadcast Receiver

The GAEN documentation states apps must register a Broadcast Receiver that handles the ACTION_EXPOSURE_STATE_UPDATED intent. This intent is broadcast when the user’s exposure status has changed. The documentation contains a recommended way of doing this, as seen in Listing 2. 
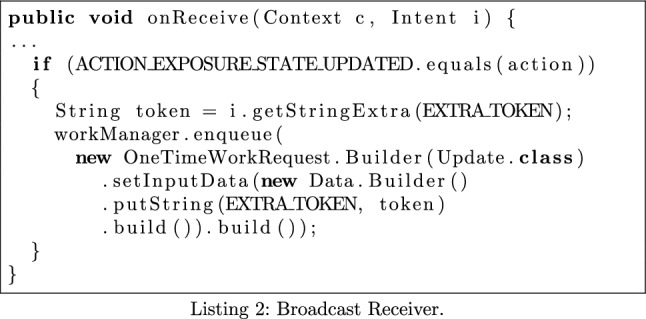

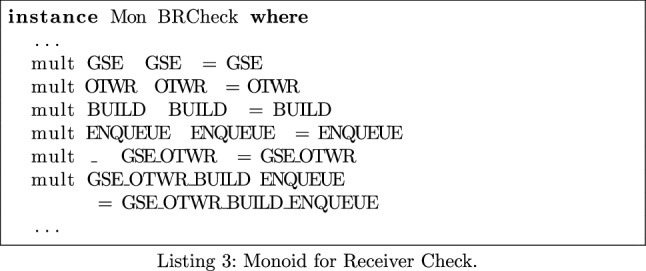




We implement a check to verify whether apps follow this recommendation. We identify the methods that compose this pattern: getStringExtra(), OneTimeWorkRequest<init>(), build() and enqueue() and replace them with the keywords **gse**, **otwr**, **build** and **enqueue**. As we are interested in a specific pattern, we do not need to define our multiplication rules fully, treating irrelevant situations as having no effect. Defining the monoid this way, we produce an element that represents the behaviour we are hoping to express: **gse_otwr_build_enqueue**. If this element is in MonSTER’s output, then the pattern is present in the app.

### Handling of Keys

We introduce another check to ensure the apps are correctly managing their keys. This consists of two parts: the handling of the TEKs and the handling of the Diagnosis Keys. For the TEKs, we verify that they are accessed only to be submitted to a central server, i.e., retrieving the keys is always followed by a network sink. For the Diagnosis Keys, we ensure that they are downloaded from a central server and then provided to the API, i.e., providing the keys is always preceded by a network source. To do this, we build a monoid consisting of the GAEN API methods getTemporaryExposureKeyHistory(), provideDiagnosisKeys() and all network sinks/sources. We encode these as **recentkeys**, **providekeys** and **network**. We also introduce an element **double_share** that allows us to see if there are multiple paths through the app that lead to TEK sharing. 
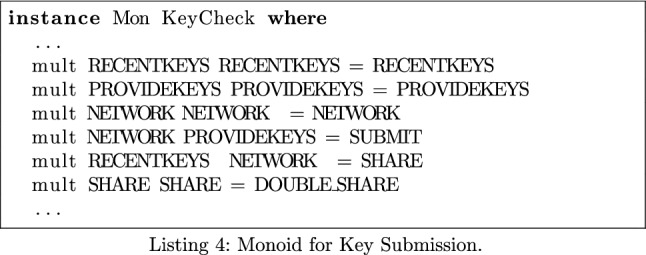


### Notifying Users of Exposure

When a user becomes potentially infected after being exposed to an infected passerby, the app should inform the user via a push notification. Again, we can modify MonSTER to test whether this happens across all of the apps tested.

We use the monoid displayed in Listing 5 to test whether a notification is created after the ACTION_EXPOSURE_STATE_UPDATED broadcast receiver is triggered. We introduce a fictional method **receive** to the start of the broadcast receiver’s onReceive() method in the call flow expression form of each app. Furthermore, we replace any methods that create a push notification—such as NotificationCompat.Builder()—with the keyword **notify**. In particular, we are hoping we see the element **receive_notify** in the output. 
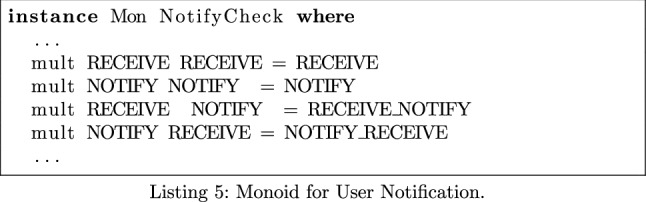


### Updating the UI Correctly

Although not mentioned in the GAEN documentation, ensuring the user interface accurately reflects the state of the GAEN client is important. For instance, if an app stopped sharing TEKs but failed to indicate this, the user would reasonably assume they have a greater level of protection against COVID-19 than in reality. Similar problems may occur with privacy-conscious users unwittingly sharing TEKs.

The UI should update regardless of the entrypoint into the app. This problem can easily be represented in MonSTER by encoding each stage of the Android app lifecycle as a method. These methods can then call the methods of other stages in the lifecycle that are immediately reachable. This technique embeds all potential paths through the Android app lifecycle in the app’s call flow expression, allowing us to check if a property occurs in any possible path.

Following these preparations, we build our monoid as before. We are hoping to see a call to isEnabled() followed by a call to any function that changes the UI, which we treat as a single class of methods. We replace these with **enabled** and **ui_change** respectively. The desired pattern is represented by the element **updated**. 
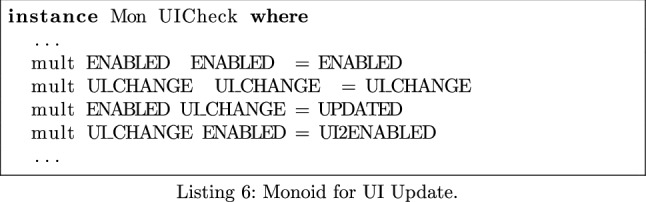


## Results

In this section, we discuss the results of the three stages of our analysis.

### Manual Analysis

All of the apps tested could be described as wrappers around the GAEN API of various sizes and complexity, with the exception of *Covid-Safe-Paths* which has a GPS tracing mode. Almost all the activities of these apps are static and there are few ways the user can input arbitrary data; users can often only enter a random identification number to confirm they have been tested. There is no link between the user’s identity and the identification number, which is provided in person at a medical centre.

#### Permissions and Services

In many of our apps, there is a failure to accurately convey the services and permissions needed for the GAEN client to operate. On Android, apps must request the Internet and Bluetooth permissions. They do not need any location permissions. However, Android requires Bluetooth, location and network *services* are active for the GAEN to function. In Google Play reviews, several users express confusion over this distinction, questioning why an app needs location services when it claims not to be tracking users.

During our manual analysis, we found that several apps exacerbate this problem by failing to properly check whether the needed services are running when turning on exposure notifications, namely *Apturi Covid*, *Corona-Warn-App*, *Covid Tracker App* and *Protect Scotland*. All of these apps indicate exposure notifications are active when the GAEN client is blocked at an OS level due to some services not running. *Corona-Warn-App* and *Protect Scotland* trigger a notification asking the user to activate the required services, but this is unreliable and can be dismissed. Otherwise, users are only informed of this problem in their phone’s settings menu. This could easily impact the effectiveness of the GAEN protocol and user safety as user’s may be misinformed to their level of protection against COVID-19.

#### Individual App Comments

We include some comments for a subset of seven tested apps. These apps fulfill one of the following requirements: either problems with their functionality were uncovered during out analysis process or significant changes were made to their design between the first and second round of our analysis.

**Apturi Covid** During the first round of investigations, we found that if the user hands the responsibility of managing the GAEN client from this app to another app, *Apturi Covid* still indicates it is seeking nearby keys when, in actuality, it isn’t. If the user then deactivates exposure notifications in the other app, *Apturi Covid* still indicates it is actively working, when no exposure notifications are being sent. We think that this is a significant safety problem, as a user could be misled into believing they were protected by the app when nothing was happening. We notified the developers of *Apturi Covid* of this and received a response stating they would fix this issue. As highlighted by the MonSTER test, the problem was caused by a failure to check whether the exposure notification service was enabled through every path in the Android Lifecycle. During the second round of checks, we found that this problem still existed in the updated version of *Apturi Covid*.

**Corona-Warn-App** During the first round of checks, this is one of three apps whose functionality was questionable. When a user switches between contact tracing apps, this app throws a Java exception error. The error states that the GAEN API is not active, although it actually is; it’s just not being managed by *Corona-Warn-App*. Although minor, this error together with other problems discussed in this section may exacerbate confusion about the GAEN client’s functionality.

The complexity of this app increased dramatically between the two versions that we tested, with the source code expanding by more than 700% over the course of the year. Although some of this increase is due to additional features—such as allowing users to set up a QR code for others to scan during gatherings for better tracking and allowing users to upload a digital copy of their EU Digital Vaccine Certificate—much of it is caused by increased design complexity. This expanded code size, alongside the introduction of libraries that are not supported by MonSTER, made it impossible to run some of the bespoke checks.

**Covid-Safe-Paths** During the first round of analysis, this app had two distinct functionalities, GPS tracing and BLE tracing. Therefore, its attack surface is larger than other apps tested and it collected more sensitive information. Although users could delete their GPS location history from the app, there was no way from within the app to turn off this feature.

Since the first round of the analysis, both Google and Apple have enforced stricter guidelines on the functionality that an app can have whilst maintaining access to the GAEN API, banning developers from tracking users. As a result, PathCheck, the organisation behind *Covid-Safe-Paths*, ceased development, and began focusing their efforts on developing a new contact-tracing app, *PathCheck*, that relies exclusively on the GAEN framework and bears little resemblance to the original *Covid-Safe-Paths*.

**Covid Tracker App** This app was donated to the Linux Foundation Public Health group; modified versions of it may be developed for other countries. The Linux Foundation will take care of the app’s maintenance. The app allows the user to supply a phone number, but this number is stored on the phone and is only shared with the health authority if the user tests positive and provides permission. *Covid Tracker App* collects anonymised metric data, such as whether the user deleted the app during the onboarding process, but users must opt-in to this service. The second version of *Covid Tracker App* that we tested allows the user to store a copy of an EU Digital COVID Certificate on their phone by scanning a QR code.

**NHS Test & Trace. (England/Wales)** *NHS Test & Trace* was the first GAEN app that allowed users to scan QR codes outside of public spaces to provide the app with a rough user location and, thus, this functionality was available during the first round of analysis. However, this analysis was limited, because a beta key was necessary to access the app’s full functionality, which we did not get access to.

**Stopp Corona.** As with *Apturi Covid*, switching between apps or toggling the GAEN client via the phone’s settings causes problems with this app. As tracing apps act passively in the background, we feel that this could be a significant problem as a user rarely interacting with *Stopp Corona* app may be inadvertently unprotected. Following the first round of analysis, we notified the developers of *Stopp Corona* of this but received no response. The problem remained unfixed during the second round of analysis.

**SwissCovid.** *SwissCovid* is produced by the researchers behind DP3T and can be seen as its reference implementation. We found the information and notifications shown to the user to be of a high standard compared to other apps, even warning users of the risk of linkage attacks when submitting their keys. Like other apps, between the first and second rounds of the analysis, *SwissCovid* was updated to allow users to scan QR codes to register their location with a venue.

### General Static Analysis

**Table 2:** MobSF summary of Apps in Table 1 as at July 2020

App name	Comp.	Additional permissions	Certificate	Trackers	Score
Apturi Covid	4	WL	v1, v2	2	65
Corona Warn	3	WL CA	v1, v2, v3	0	50
Safe Paths	11	WL NL GL DS AA DS AR AA ST AT	None	1	5
CovidShield	4	WL AW	None	0	45
Covid Tracker	4	None	v1, v2	0	30
Immuni	3	WL	v1, v2	0	35
NHS Test & Trace	6	WL CA	v1, v2, v3	0	60
Protect Scotland	6	None	v1, v2, v3	0	30
ProtegoSafe	5	WL	v1, v2	2	70
Stop-Covid-19	3	WL	v1, v2, v3	3	90
Stopp Corona	3	WL BA NL LB	v1, v2	0	35
SwissCovid (DP3T)	3	WL	v1, v2	0	45

Key: *WL* Wake Lock, *BA* Bluetooth Admin, *CA* Camera, *AW* Alert Window, *NL* Network Location, *GL* GPS Location, *LB* Location in Background, *DS* Device Sync, *AR* Activity Recognition, *AA* Account Authentication, *ST* Device Storage, *AT* Access OS Task List

We outline the results of the MobSF scans in Tables 2 and 3 which detail our static analysis in 2020 and 2021, respectively. These tables contain the number of exported components, the number of potentially dangerous permissions requested, the certificate signing schemes used, the number of tracking libraries used and the code score—a score MobSF generates to surmise the code quality, ranging from 0 (worst) to 100 (best).

The OWASP Mobile Security Testing Guide (MSTG)^5^ suggests these checks are relevant:

- exported components form an attack surface that may be exploited by malware. Thus, exported components could cause future vulnerabilities;
- tracking libraries introduce potential privacy violations of user data;
- insufficient signing schemes prevent developers from rotating their signing keys;
- additional permissions represent the capability for the phone to access user data or undertake “risky” actions.

For the number of exported components, *Covid Safe Paths* exceeds the other examples by some margin, demonstrating a concerning attack surface.

Due to the privacy-focus of these apps, most use no tracking libraries. Of the apps that use tracking libraries, these consist of Google Crashlytics, Firebase Analytics and, in the case of the earlier version of *Stop-Covid-19*, Google Admob. All of these libraries allow for the harvesting of information that is tangential to the operation of the GAEN API and could harm user privacy.

For APKs downloaded from the Play Store, the certificate signing schemes used are shown. MSTG recommends using all three schemes in apps that target modern Android SDK levels. During the first round of analysis, only 3 apps do this.

**Table 3:** MobSF summary of Apps in Table 1 as at August 2021

App name	Comp.	Additional permissions	Certificate	Trackers	Score
Apturi Covid	6	WL	v1, v2, v3	2	80
Corona Warn	3	WL CA	v1, v2, v3	0	30
Covid Tracker	7	CA	v1, v2, v3	0	45
Immuni	4	WL	v2, v3	0	30
NHS Test & Trace	7	None	v1, v2, v3	0	40
Protect Scotland	7	None	v1, v2, v3	0	40
ProtegoSafe	7	WL	v1, v2	2	90
Stop-Covid-19	2	WL	v1, v2, v3	2	100
Stopp Corona	5	WL	v1, v2	0	75
SwissCovid (DP3T)	6	WL	v1, v2	0	65

Key: *WL* Wake Lock, *BA* Bluetooth Admin, *CA* Camera, *AW* Alert Window, *NL* Network Location, *GL* GPS Location, *LB* Location in Background, *DS* Device Sync, *AR* Activity Recognition, *AA* Account Authentication, *ST* Device Storage, *AT* Access OS Task List

For apps using GAEN, the minimum required permissions are Bluetooth and Internet; some apps request much more than is necessary, particularly *Covid Safe Paths* and *Stopp Corona*. The version of *Covid Safe Paths* analysed includes GPS tracking functionality, which accounts for GL, but the other permissions seem unnecessary when contrasted with other apps. Similarly, *Stopp Corona* requests the usage of Bluetooth Admin, which is strongly discouraged by the GAEN documentation, and location services and is thus over-privileged. Permission creep is a well-established problem in Android [17] and the principle of least privilege is considered good practise by MSTG. *Corona-Warn-App*, *NHS Test & Trace* and the later versions of *Covid Tracker* require the use of the phone’s camera (CA) as these apps utilise QR-code scanning.

As most functionality of the GAEN framework is provided by the API, apps only need to be a wrapper around it, preferably as thin as possible. Any additional functionality increases the risk of security, privacy or functionality issues. This is reflected in the code score of each app which, as can be seen by cross-referencing Table 1 with Table 2, is inversely correlated with the code size; *Covid-Safe-Paths* has the worst score and is largest, whilst *Stop-Covid-19* has the best score and is smallest.

Contrasting Table 2 with Table 3, we observe that most of the contact tracing apps have been improved according to several factors, including decreasing the number of exported components, decreasing requests for additional dangerous permissions, upgrading their certificate signing schemes and decrease the number of long running trackers. These changes significantly improve the security and privacy of an application by reducing the apps overall attack surface and by decreasing the permission and privilege level of the applications, reducing an adversaries capabilities following a successful attack. These improvements in our sample of contact tracing apps shows that the general populace is demanding more secure apps to protect their privacy and that developers have started to update their apps towards these ends. The methodology proposed in this work aims to provide developers with an immediate, customisable process to discover potential problems and thus allow them to rectify those issues. In general, this should help to improve the overall security and privacy considerations of essential contact tracing apps.

### Bespoke Static Analysis

**Table 4:** Results of our MonSTER checks (2020)

App name	1	2	3	4	5
Apturi Covid	$\checkmark$	$\checkmark$	$\checkmark$	$\checkmark$	X
Covid Tracker App	$\checkmark$	$\checkmark$	–	$\checkmark$	$\checkmark$
Corona-Warn-App	$\checkmark$	$\checkmark$	$\checkmark$	$\checkmark$	$\checkmark$
CovidShield	$\checkmark$	$\checkmark$	–	–	–
Covid Safe Paths	$\checkmark$	$\checkmark$	$\checkmark$	X	–
Immuni	$\checkmark$	$\checkmark$	$\checkmark$	–	$\checkmark$
Protect Scotland	$\checkmark$	$\checkmark$	–	$\checkmark$	$\checkmark$
ProtegoSafe	X	$\checkmark$	$\checkmark$	–	$\checkmark$
NHS Test & Trace	$\checkmark$	$\checkmark$	$\checkmark$	$\checkmark$	$\checkmark$
Stop-Covid-19	X	X	$\checkmark$	$\checkmark$	$\checkmark$
Stopp Corona	$\checkmark$	$\checkmark$	$\checkmark$	$\checkmark$	X
SwissCovid (DP3T)	$\checkmark$	$\checkmark$	$\checkmark$	$\checkmark$	$\checkmark$
Misconfigured App	X	X	X	X	X

The output of MonSTER is a list of tuples containing a method name and the monoid elements that can be reached from that method. We confirm the existence of a pattern in an app’s source code by the existence of the monoid element representing that pattern in the output. We summarise the results for the two rounds of analysis in Tables 4 and 5.

**Check 1-Starting Tracing in a Suitable Manner** All apps tested met this requirement except for *ProtegoSafe* and *Stop-Covid-19*, indicating neither app checks whether the Exposure Notification client is running before starting.

**Check 2-Registering a Broadcast Receiver** All apps tested met this requirement, except *Stop-Covid-19*. Of those that passed, all but one followed the implementation listed in the Google documentation exactly. The app that failed was *Stop-Covid-19* which registered a broadcast receiver but did not do so in the manner described in the documentation.

**Check 3-Handling of Temporary Keys** We could not perform this test on *Covid Tracker App*, *CovidShield* and *Protect Scotland* as parts of this process are coded in TypeScript; similarly, we could not test *Corona-Warn-App* during the second round of analysis due to unsupported libraries. For all other apps, we found that all calls to getTemporaryExposureKeys() are followed by a network sink and all calls to provideDiagnosisKeys() are preceded by a network source. We also ensure that keys are sent to a single sink when submitted. We surmise that if a user is presented with the option to share their keys, all apps tested submit them to only one Diagnosis Server. Similarly, we conclude that after retrieving the Diagnosis Keys from the server, these apps correctly provide them to the API.

**Check 4-Notifying Users of Exposure** Again, we could not run this check on *CovidShield* as it is largely written in Typescript. Furthermore, the heavy use of dependency injection or unsupported libraries in *Immuni*, *ProtegoSafe* and *Corona-Warn-App* (during the second round of analysis) limits MonSTER’s ability to generate meaningful call flow expressions which hinders its ability to analyse these apps. Of the apps properly tested, only *Covid-Safe-Paths* failed.

**Check 5-Updating the UI Correctly** *Stopp Corona* and *Apturi Covid* failed this check during both rounds of our analysis. The logic for updating the UI in these apps is handled in the onCreate() method of the main landing page instead of onResume(). Thus, one can activate exposure notifications, close the app and turn them off—either in the phone’s settings or using another contact tracing app—and neither app will update the UI, instead incorrectly informing the user that the app is contact tracing. As discussed in the section “Individual App Comments”, we informed the developers of *Apturi Covid* of this and received confirmation that the problem would be fixed. However, the app still failed the check. Manual testing confirmed this behaviour.

As seen from Table 3 and “General Static Analysis”, the results generated by MobSF, although worthwhile from a security perspective, reveal nothing about the usage of the GAEN framework. In contrast, the bespoke analysis with MonSTER allows us to generate strong guarantees about the functionality of the apps and their adherence to the GAEN requirements.

Although MonSTER requires more effort to produce customised checks, its advantages over general static analysis are clear. MonSTER’s call flow checking allows the user to fine-tune the properties to be checked that are unique to a given app or set of apps. Such properties can then be checked repeatedly throughout the app’s development and release.

**Table 5:** Results of our MonSTER checks (2021)

App name	1	2	3	4	5
Apturi Covid	$\checkmark$	$\checkmark$	$\checkmark$	$\checkmark$	X
Covid Tracker App	$\checkmark$	$\checkmark$	–	$\checkmark$	$\checkmark$
Corona-Warn-App	$\checkmark$	$\checkmark$	–	–	$\checkmark$
Immuni	$\checkmark$	$\checkmark$	$\checkmark$	–	$\checkmark$
Protect Scotland	$\checkmark$	$\checkmark$	–	$\checkmark$	$\checkmark$
ProtegoSafe	X	$\checkmark$	$\checkmark$	–	$\checkmark$
NHS Test & Trace	$\checkmark$	$\checkmark$	$\checkmark$	$\checkmark$	$\checkmark$
Stop-Covid-19	X	X	$\checkmark$	$\checkmark$	$\checkmark$
Stopp Corona	$\checkmark$	$\checkmark$	$\checkmark$	$\checkmark$	X
SwissCovid	$\checkmark$	$\checkmark$	$\checkmark$	$\checkmark$	$\checkmark$

## Limitations

MonSTER is a prototype designed to explore our methodology and thus has issues. One pitfall is scalability; larger apps take far longer to analyse. We can mitigate this problem by excluding irrelevant code from the analysis. Moreover, we can only analyse patterns that appear in the app’s bytecode, i.e., those written using Java or Kotlin. Finally, some programming constructs that rely on generated methods, such as Dependency Injection libraries and coroutine support, limit MonSTER’s ability to build accurate call flow expressions, requiring manual fixing.

When evaluating apps for this paper, we found few we could properly analyse. Many tracing apps using the GAEN API are not open source and these all used code obfuscation. We believe that this is counter-productive to the goals of the GAEN as the end-user has little guarantee of the app’s capabilities and whether it has faithfully implemented the protocol. To instill greater trust in end-users that the apps are working as intended, providing a public verification method, such as open-source code or third-party audits, would be advantageous. In these times, accurately functioning contact tracing should take precedence over intellectual property.

## Related Work

Unlike our work which focuses on correct implementation, most current research on contact tracing apps focuses on the design of the underlying frameworks, particularly with respect to privacy. Cho et al. define three notions of privacy for contact tracing apps: privacy from snoopers, contacts, and the authorities [18]. They note that some information will always be revealed and simple attacks can always be performed; therefore, an acceptable level of privacy should be defined with respect to these three parties. Gvili analyzes privacy issues with the GAEN framework and proposes attacks that would hinder its effectiveness, such as relay and replay attacks [19]. Similarly, Magklaras et al. assess the weaknesses of published tracing frameworks [20]. Some research does focus on app implementations, but at a higher level compared to us. Samhi et el. provide a categorisation of existing apps on Google Play related to COVID-19, but do not analyse apps individually [21].

Following our first round of analysis, research evaluating the security and privacy issues of contact tracing apps independently from the underlying framework increased. Both Hatamian et al. [22] and Kouliaridis et al. [23] employed a similar analysis to ours, combining static, dynamic and manual analysis to evaluate the security and privacy properties of contact tracing apps. However, their analysis does not include a bespoke static analysis stage as ours does and they do not check whether apps adhere to the GAEN documentation. Similarly, Sun et al. [24] produce a tool titled *COVIDGuardian* that incorporates static and data flow analysis to determine security and privacy weaknesses. Although targeted towards contact tracing apps, COVIDGuardian lacks the flexibility of MonSTER which allows for bespoke rules to be verified regardless of domain.

There are many static analysing tools for the Android platform. Li et al. [25] identified over 100 such tools. Unlike MonSTER, the majority of these tools establish that an app is secure using a generalised ruleset. For instance, the MobSF [14] and QARK [15] work by analysing decompiled code and flagging bad programming practises that may lead to security issues, such as the existence of logging or API keys. Some tools are more specific but lacking the customisation of MonSTER; for instance, taint analysis research has led to tools such as FlowDroid [26], designed to ensure sensitive information cannot be exfiltrated from an app. MonSTER can also be seen as a static analogue of dynamic analysis call tracing, utilised by tools such as DroitMat [27] and DroidTrace [28]. However, both of these tools focus on identifying malware, rather than functionality properties like MonSTER.

## Retrospective and Conclusion

### Retrospective

Between our first and second round of analysis, the landscape of contact tracing apps has changed considerably as these apps adapt to the limitations of digital contact tracing. For instance, Leigh et al. [9] found the reliance on BLE to be lacking as it failed to detect exposures in indoor spaces. This manifested during the production of the first NHS app, preventing its release. Leigh et al. therefore suggested supplementing the protocol by introducing a stage where the user can scan a QR code at the entrance to an indoor area to indicate their location, an addition that has been adopted by several of the apps that we tested. Furthermore, we see the incorporation of digital *Vaccine Passports* in the current versions of *Covid Tracker App*, *Corona Warn App* and *Immuni*, with other apps planning to introduce similar functionality. Obviously, this introduces new privacy and functionality concerns not considered by our MonSTER checks.

Despite being available for download for over a year at the time of writing, the evidence that contact tracing apps meaningfully reduce the spread of infection remains thin. The first impediment to their effectiveness is uptake; although many counties saw a sizable percentage of their population use these apps—for instance, a report by privacy watchdog Liberties found that *NHS Test & Trace* and *COVID Tracker* have been downloaded by 36% and 49% of their respective target populations as of February 2021 [29]—other apps achieved considerably less adoption. Again, Liberties report that, as of February 2021, only 2% of the Croatian population had downloaded *Stop-Covid-19*, released in July 2020. Clearly, the release of contact tracing apps necessitates health authorities to assuage privacy concerns and encourage widespread adoption, with some research demonstrating the effectiveness of monetary incentives.

However, even in populations where contact tracing apps saw high uptake, their efficacy is questionable. In an analysis of the effectiveness of *SwissCovid* in the canton of Zurich, Menges et al. [30] estimated that, in the month of September 2020, the app triggered notifications for 1374 individuals. Of these, 722 users called the quarantine helpline, 170 were instructed to quarantine and 30 tested positive for COVID-19. With only roughly 2% of notified users testing positive for COVID-19—with no evidence that they were infected due to the close contact event that triggered the notification—the extent to which contact tracing apps reduce the strain on manual contact tracing systems is potentially minimal. Furthermore, in the case of *NHS Test & Trace*, the specific threshold values chosen to notify users of close contacts were too sensitive, resulting in an exponential increase in the number users told to self-isolate by the app, peaking at over 600,000 a week [31]. This resulted in the temporary closure of many business who could not operate due to the large percentage of staff required to quarantine, despite the fact that the overwhelming majority of these close contacts were likely false positives. As one of the stated goals by the GAEN project is to reduce the economic strain of the pandemic, such an outcome seems counter-productive.

### Conclusion

This paper presented an analysis into the functionality, security and privacy of contact tracing apps using a methodology involving manual, general static and bespoke static analysis. For the bespoke case, we present MonSTER, a lightweight, static analysis tool that can detect the existence of patterns of Android app behaviour in a customisable way, as general static analysis tools were not sufficient. Using this process, we verified that many contact tracing apps adhered to the GAEN API’s recommended usage. However, we found failings in tested versions of some apps that could impact user safety or security, namely *Covid-Safe-Paths*, which failed to adhere to design practises that minimise user risk, *Apturi Covid* and *Stopp Corona*, which failed to correctly inform users of the status of the GAEN client. For future work, we mention that MonSTER’s generation of call flow expressions from an app’s bytecode could be improved to capture more programming constructs, such as coroutines.

## Footnotes

1 https://github.com/glo-fi/MonSTER.

2 https://github.com/glo-fi/GAEN-Contact-Tracing-Apps.

3 During our first round of analysis, *Protect Scotland* was only partially open-sourced.

4 https://github.com/google/exposure-notifications-android.

5 https://github.com/OWASP/owasp-mstg/.

## References

[1] Google. Google/Apple exposure notifications: Android API documentation PDF. Version 1.3.2. 2020. https://web.archive.org/web/20200603200341/https://static.googleusercontent.com/media/www.google.com/en//covid19/exposurenotifications/pdfs/Android-Exposure-Notification-API-documentation-v1.3.2.pdf. Accessed 04 Aug 2020.

[2] Troncoso C, Payer M, Hubaux J-P, Salathé M, Larus J, Bugnion E, Lueks W, Stadler T, Pyrgelis A, Antonioli D, et al. Decentralized privacy-preserving proximity tracing. 2020. arXiv preprint. arXiv:2005.12273.

[3] Wan Z, Liu X. ContactChaser: a simple yet effective contact tracing scheme with strong privacy. Cryptology ePrint Archive, Report 2020/630. 2020. https://eprint.iacr.org/2020/630.

[4] Amnesty. Bahrain, Kuwait and Norway contact tracing apps among most dangerous for privacy. 2020. https://www.amnesty.org/en/latest/news/2020/06/bahrain-kuwait-norway-contact-tracing-apps-danger-for-privacy/. Accessed 04 Aug 2020.

[5] Anomali. Anomali threat research identifies fake COVID-19 contact tracing apps used to download malware that monitors devices, steals personal data. 2020. https://www.anomali.com/blog. Accessed 10 Sept 2020.

[6] ESET. New ransomware posing as COVID-19 tracing app targets Canada. 2020. https://www.welivesecurity.com/2020/06/24/. Accessed 10 Sept 2020.

[7] Bortolozzo M, Centenaro M, Focardi R, Steel G. Attacking and fixing PKCS#11 security tokens; 2010. p. 260–9.

[8] TCN. TCN coalition. 2020. https://web.archive.org/web/20200817060508/https://tcn-coalition.org/. Accessed 5 Sept 2022.

[9] Leith DJ, Farrell S (2020). Coronavirus contact tracing: evaluating the potential of using bluetooth received signal strength for proximity detection. Comput Commun Rev.

[10] PePP-PT. Pan-European privacy-preserving proximity tracing. 2020. https://www.pepp-pt.org/. Accessed 04 Aug 2020.

[11] Sadowski C, Aftandilian E, Eagle A, Miller-Cushon L, Jaspan C (2018). Lessons from building static analysis tools at Google. Commun ACM.

[12] Ayewah N, Pugh W, Hovemeyer D, Morgenthaler JD, Penix J (2008). Using static analysis to find bugs. IEEE Softw.

[13] Kleinman Z. NHS Covid-19: app issue fixed for people who test positive. 2020. https://www.bbc.com/news/technology-54307526. Accessed 06 June 2022.

[14] Abraham A, Schlecht D, Dobrushin M, Nadal V. Mobile security framework (MobSF). 2016. https://github.com/MobSF. Accessed 5 Sept 2022.

[15] LinkedIn. Quick Android review kit (QARK). 2015. https://github.com/linkedin/qark. Accessed 5 Sept 2022.

[16] Desnos A, et al. Androguard. 2015. https://github.com/androguard/androguard. Accessed 5 Sept 2022.

[17] Vidas T, Christin N, Cranor L. Curbing android permission creep. In: Proceedings of the Web, vol. 2; 2011. p. 91–6.

[18] Cho, H, Ippolito, D, Yu, Y. Contact tracing mobile apps for COVID-19: Privacy considerations and related trade-offs. arXiv preprint. 2020. arXiv:2003.11511.

[19] Gvili, Y. Security analysis of the COVID-19 contact tracing specifications by Apple Inc. and Google Inc. Cryptology ePrint Archive. 2020.

[20] Magklaras G, Bojorquez LNL, Clarke N, Furnell S (2020). A review of information security aspects of the emerging COVID-19 contact tracing mobile phone applications. Human aspects of information security and assurance.

[21] Samhi J, Allix K, Bissyandé TF, Klein J (2020). A first look at android applications in Google Play related to Covid-19. Empir Softw Eng.

[22] Hatamian M, Wairimu S, Momen N, Fritsch L (2021). A privacy and security analysis of early-deployed COVID-19 contact tracing Android apps. Empir Softw Eng.

[23] Kouliaridis V, Kambourakis G, Chatzoglou E, Geneiatakis D, Wang H. Dissecting contact tracing apps in the Android platform. PloS One. 2021;16(3). 10.1371/journal.pone.0251867 PMC8121305 33989350

[24] Sun R, Wang W, Xue M, Tyson G, Camtepe S, Ranasinghe DC. An empirical assessment of global COVID-19 contact tracing applications. In: 2021 IEEE/ACM 43rd international conference on software engineering (ICSE). IEEE; 2021. p. 1085–97.

[25] Li L, Bissyandé TF, Papadakis M, Rasthofer S, Bartel A, Octeau D, Klein J, Traon L (2017). Static analysis of Android apps: a systematic literature review. Inf Softw Technol.

[26] Arzt S, Rasthofer S, Fritz C, Bodden E, Bartel A, Klein J, Le Traon Y, Octeau D, McDaniel P (2014). Flowdroid: precise context, flow, field, object-sensitive and lifecycle-aware taint analysis for Android apps. ACM SIGPLAN Not.

[27] Wu D-J, Mao C-H, Wei T-E, Lee H-M, Wu K-P. Droidmat: Android malware detection through manifest and API calls tracing. In: 2012 Seventh Asia joint conference on information security. IEEE; 2012. p. 62–9.

[28] Zheng M, Sun M, Lui JC. DroidTrace: a ptrace based Android dynamic analysis system with forward execution capability. In: 2014 International wireless communications and mobile computing conference (IWCMC). IEEE; 2014. p. 128–33.

[29] Civil Liberties Union for Europe. COVID-19 contact tracing apps in the EU. 2021. https://www.liberties.eu/en/stories/trackerhub1-mainpage/43437. Accessed 20 Sept 2021.

[30] Menges D, Aschmann HE, Moser A, Althaus CL, von Wyl V. The role of the SwissCovid digital contact tracing app during the pandemic response: results for the Canton of Zurich. medRxiv preprint; 2021.

[31] Plummer R. ‘Pingdemic’ dents UK economic growth in July. 2021. https://www.bbc.co.uk/news/business-58502593. Accessed 06 June 2022.

